# Autosomal dominant mutation of MSX1 gene causing tooth and nail syndrome

**DOI:** 10.11604/pamj.2020.36.229.23019

**Published:** 2020-07-29

**Authors:** Mohammed Najmuddin, Safeena Abdul Khader Saheb, Abdulrahman Nahi Alharbi, Fahad Mohammed Alsobil, Chitra Jhugroo, Aftab Ahmed Khan, Darshan Devang Divakar, Sachin Naik, Sanjeev Balappa Khanagar

**Affiliations:** 1 Oral Medicine and Maxillofacial Radiology, Department of Maxillofacial Diagnostic Sciences, College of Dentistry, Jazan University, Jazan, Kingdom of Saudi Arabia,; 2 Division of Orthodontia, Department of Preventive Dental Sciences, College of Dentistry, Jazan University, Jazan, Kingdom of Saudi Arabia,; 3 General Dentist, Ministry of Health, Qaseem, Kingdom of Saudi Arabia,; 4 General Dentist, Ministry of Health, Riyadh, Kingdom of Saudi Arabia,; 5 Dental Biomaterials Research Chair, Dental Health Department, College of Applied Medical Sciences, King Saud University, Riyadh 11433, Kingdom of Saudi Arabia,; 6 Dental Public Health, Preventive Dental Science Department, College of Dentistry, King Saud bin Abdulaziz University for Health Sciences, Riyadh, Saudi Arabia; King Abdullah International Medical Research Center, Ministry of National Guard Health Affairs, Riyadh, Saudi Arabia

**Keywords:** Partial anodontia, tooth and nail syndrome

## Abstract

Tooth and Nail Syndrome or Nail Dysplasias with Hypodontiaor Witkop´s Syndrome is an autosomal dominant condition present at birth and improves by age. An early diagnosis is essential to avoid future functional, aesthetic, and psychological problems. Here we report two classic cases with brief clinical, radiological and genetic investigation along with a brief review of literature.

## Introduction

Witkop´s tooth and nail syndrome is an uncommon autosomal dominant disorder characterized by nail dysgenesis and hypodontia with morphological changes in the teeth [1-6]. It is brought about by a single duplicate of a responsible gene that is most often taken from a parent who is affected as well by the disease [5]. This condition differs from hypohidrotic form of Ectodermal dysplasia as there is no involvement of sweat glands and hairs [3, 4]. It is very rare, and the incidence is reported to be 1-2: 10000, equally affecting both male and female [1, 2, 7, 8]. The affected individuals often have widely spaced, conical shape teeth with narrow crowns [5]. Nails exhibit koilonychia (spoon-shaped), onychorrhexis (easily breakable), and they are generally slow-growing. These defects are usually alleviated with age affecting toenails more severely than fingernails [1, 4, 5, 7]. These individuals do not have any specific facial looks. The inborn missing teeth can gravely impair the patient's bodily and psychologically, mainly during teenage [3]. There are two types of tooth and nail syndrome (Witkop syndrome and Fried syndrome), which differ in the pattern of inheritance [2]. The gene MSX1 is responsible for tooth and nail syndrome, which was recognized in 2001. This gene is identified as significant in tooth formation and due to the mutation of an inoperative protein results in Witkop´s tooth and nail syndrome [5].

## Patient and observation

**Figure 1:**
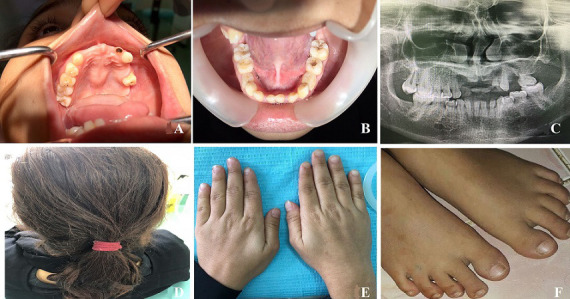
A) maxillary arch showing retained deciduous teeth, missing permanent teeth and palatal tori; B) mandibular arch showing retained deciduous teeth, missing permanent teeth; C) panoramic radiograph showing generalised absence of permanent tooth bud in both arch; D) thinning and splitting of hair; E) thin and brittle fingernails; F) toenails showing ridging

**Case 1:** an 18-year-old unmarried school going female patient, reported to the outpatient Department of Oral Medicine and Maxillofacial Radiology with a chief complaint of unerupted upper front permanent teeth. She was moderately built and nourished with a short stature, she revealed no significant systemic illness with no deleterious and par-functional habits. On general physical examination the patient was well oriented, conscious she had very thin eyebrows, there was no history of heat intolerance or problems in sweating, she had spoon-shaped slow-growing small fingernails with onychorrhexis with deformed toenails. History revealed that her nails had improved with age but were small and discolored at birth. The patient´s family history revealed that her younger sister has a similar problem. On intra oral examination generalized missing permanent teeth with retained deciduous teeth with severe attrition was noticed. Generalized pit and fissure caries was noticed on all the posterior teeth one deep caries was noticed with left maxillary anterior teeth. Maxillary palatine tori noticed with poor gingival and periodontal condition, oral mucosa was normal (Figure 1).

**Investigation:** panoramic radiograph confirmed the clinical data, showed periapical infection with right maxillary anterior teeth, generalised absence of permanent tooth bud in both maxillary and mandibular arch was shocking (Figure 1).

**Figure 2:**
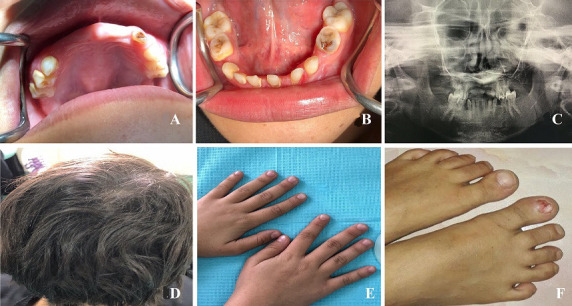
A) maxillary arch showing retained deciduous teeth, missing permanent teeth and palatal tori; B) mandibular arch showing retained deciduous teeth, missing permanent teeth; C) panoramic radiograph showing generalised absence of permanent tooth bud in both arch; D) thinning of hair; E) thin and brittle fingernails; F) toenails showing ridging and koilonychia

**Case 2:** a 14-year-old school going girl, sibling of the case 1, was examined after clinical examination of her elder sister, she was moderately built and nourished for her age, she revealed no significant systemic illness with no deleterious and par-functional habits. On general physical examination the patient was well oriented and conscious, she had similar spoon-shaped slow-growing small fragile fingernails with onychorrhexis with deformed toenails like her elder sister which was better compared to before. On intra oral examination generalized missing permanent teeth with retained deciduous teeth was noticed. Generalized pit and fissure caries on posterior teeth with one deep caries with right mandibular posterior teeth. Maxillary palatine tori was also common with poor periodontal condition having generalized stains and calculus, oral mucosa was normal (Figure 2).

**Investigation:** panoramic radiograph showed generalized missing of permanent tooth bud in both maxillary and mandibular arch (Figure 2). Based on family history, clinical, and radiological findings, a provisional diagnosis of partial anodontia was given considering mesoectodermal dysplasia, ectodermal dysplasia and tooth and nail syndrome (nail dysplasia with hypodontia) in differential diagnosis.

### Genetic investigation

**DNA extraction:** 5 ml of blood sample was collected from the median cubital vein of the patients. Genomic DNA was isolated from leukocytes using the standard Qiagen blood DNA minikit (Qiagen, Hilden, Germany) according to manufacturer´s protocol.

**Sanger sequencing:** in order to validate the identified MSX1 mutation, primers were designed using the Primer3 tool for MSX1 exon 1; forward primer (5' -CTGGCCTCGCCTTAT TAG C 3') and reverse primer (5'-GCCTGGGTTCTGGCTCTC 3'). A standard PCR was performed and the products were sequenced using Sanger sequencing through ABI 3500xl genetic analyzer (Thermo Fisher Scientific Waltham, MA USA).

**Result:** the present work is intended to identify the involvement of MSX1 gene variants in familial hypodontia. MSX1 is a vital gene in tooth development. The DNA sequences were analyzed and compared with DNA reference using DNASTAR alignment software (Madison, WI 53705 USA); meanwhile chromatograms were also viewed and analyzed using Sequence Scanner software (Thermo Fisher Scientific Waltham, MA USA). We have observed one point mutation on exon 1 of MSX1 (1A;c.731G > A). We observed that MSX1 is contributing to cause hypodontia. In the present study, MSX1 gene is mutated in familial hypodontia; however, the mutation of MSX1 alone might not cause phenotype change to the patient. Few other genes and mutation is the more predominant factor which might be contributing to hypodontia compared to MSX1. This can be identified by analysing whole exome or whole genome sequencing of the patient samples.

## Discussion

Witkop´s tooth and nail syndrome was reported by Witkop in 1965 as an autosomal dominant hereditary disorder affecting teeth and nails. It is a type of Ectodermal dysplasia without hairs and sweat gland involvement [9, 10]. It can be identified after the birth and gives a strong suspicion if one or more toe or fingernails are absent [4]. The most common teeth missing are maxillary incisors, canines, and second molars [3] which was noted in both of our cases. The severity of hypodontia differed in the available literature from a few missing teeth to extreme hypodontia [4]. The present cases could be categorized as one of the severe hypodontia as more than 20 permanent tooth buds are missing. Affected people also show nail deformity, which is worse in childhood and gets better over the age, which may not be evident in adulthood [2, 11] these features were also noted in our cases. Toenails are affected more than fingernails [2, 12]. Our patient had thin spoon-shaped nails, which were brittle.

Hypodontia and anodontia are frequently associated with a multitude of genetic disorders and syndromes, approximately 70. Syndromes particularly involved with ectodermal involvement are a prime circumstance for anodontia to occur, some examples of these are: Rieger's, Robinson's and focal dermal hypoplasia [13]. Three syndromes which classically have signs of anodontia are oculomandibulodyscephaly, mesoectodermal dysplasia and ectodermal dysplasia. In cases of oculomandibulodyscephaly there are no permanent teeth but there are deciduous teeth present. In mesoectodermal dysplasia the symptoms are anodontia and hypodontia. In cases of ectodermal dysplasia oligodontia is also present, hence we ruled out most conditions in our case. Similar types of Ectodermal dysplasia related to teeth and nails are Fried tooth and nail syndrome (Door syndrome) and Curry Hall syndrome. However, the absence of one or more toe or fingernails with hypodontia should be considered as Tooth and Nail syndrome [4]. A genetic evaluation can be carried out to validate the diagnosis [14] which was done in both of our cases including their parents and the results confirmed the mutation at MSX1 gene located on chromosome 4p16.

## Conclusion

The treatment of the affected individuals involves an interdisciplinary approach with prosthetic replacement, including removable, mixed implant-supported, or combination of these options [4]. It depends on the severity of the disease. However, most often, the state of hair and nails get improve when the patient gets older. Adult patients might need a psychological assessment and guidance as they might be psychologically affected due to the facial appearance [2]. In our case, fixed prosthesis or implant-supported prosthesis has been planned and kept under observation with a long term follow-up.
